# In Vivo Evaluation of Anti-Nociceptive Effects of Silver Nanoparticles

**DOI:** 10.3390/molecules27217259

**Published:** 2022-10-26

**Authors:** Shereen Morsi, Valeria Pittala, Mohammad Alqudah, Mohamed Haider, Khaled Greish

**Affiliations:** 1 Department of Physiology, Faculty of Medicine, Suez Canal University, Ismailia 8366004, Egypt; 2 Department of Drug and Health Science, University of Catania, 95125 Catania, Italy; 3 Department of Molecular Medicine, Princess Al-Jawhara Centre for Molecular Medicine, College of Medicine and Medical Sciences, Arabian Gulf University, Manama 329, Bahrain; 4 Department of Physiology, College of Medicine and Medical Sciences, Arabian Gulf University, Manama 329, Bahrain; 5 Department of Physiology and Biochemistry, College of Medicine, Jordan University of Science and Technology, Irbid 222110, Jordan; 6 Department of Pharmaceutics and Pharmaceutical Technology, College of Pharmacy, University of Sharjah, Sharjah 27272, United Arab Emirates; 7 Research Institute of Medical & Health Sciences, University of Sharjah, Sharjah 27272, United Arab Emirates

**Keywords:** silver nanoparticles, nociception, acute pain, chronic pain

## Abstract

Silver nanoparticles (AgNPs) are widely used commercially due to their antimicrobial effects. Little is known about the effect of AgNPs on neural transmission and pain response. The aim of this study was to assess the anti-nociceptive activity of AgNPs. AgNPs were prepared at 16 ug/mL, white albino rats were injected with various doses of AgNPs, and challenged using a hot-plate test and paw withdrawal latency (PWL) was measured. The chronic constriction injury (CCI) model was utilized to evaluate the pedal withdrawal reflex and tail withdrawal reflex. An electrophysiological study was conducted utilizing colon longitudinal muscle strips. AgNPs increased the latency of PWL in a dose-dependent matter over the duration of 6 h. The paw withdrawal threshold in animals with CCI significantly increased after AgNPs administration. In isolated colon longitudinal muscle strips, AgNPs significantly reduced the colonic migrating motor complexes (MMCs) and contraction. This action was completely reversed after removing the AgNPs and adding acetylcholine to the preparation. In this study, AgNPs showed significant anti-nociception properties. To our knowledge, this is the first report to describe this pharmacological action of AgNPs.

## 1. Introduction

Nociceptive pain is caused by stimuli that damage body tissues, such as severe heat, intense cold, harsh physical injury, and various chemical irritants [1]. Thin myelinated A-δ and unmyelinated C-fibers carry heat, mechanical and chemical, whereas C-fibers carry cold stimuli [2]. Under certain conditions, pain stimuli can recruit the low threshold A-β fibers [1]. The ion channels involved in pain transmission include the voltage gated sodium channels, the nonspecific transient receptor potential (TRP) with all its subtypes (TRPV1-4, TRPA1, TRPM8), voltage-operated calcium channels, and voltage-gated potassium channels [3]. These ion channels are involved in all the steps of pain sensation, including resting membrane potential, depolarization, repolarization, frequency of firing, and neurotransmitter release [4,5]. Pain is a common postoperative symptom following surgical procedures. Local anesthetics have been used to provide regional pain relief for the management of postoperative acute and chronic pain [6]. Following their cellular uptake, local anesthetics block the voltage-gated sodium channels in neuronal plasma membranes, inhibiting the occurrence of the rapid early depolarization phase of an action potential. This results in a temporary blockade in nerve fiber conduction, preventing the transmission of nociceptive signals in a major nerve trunk [7]. Despite all the benefits in acute and chronic pain management, bolus injections of commonly used local anesthetics, such as lidocaine, tetracaine, and procaine are limited by their relatively short duration and fast absorption through the surrounding tissues, leading to sharp fluctuating levels of plasma drug concentration. In addition, frequent injections are difficult to maintain and may result in poor compliance [8,9]. Nanotechnology has gained attention as a promising approach for the development of potential diagnostic and therapeutic tools for the management of cancer and other disease conditions [10,11]. The development of various types of nanoparticles (NPs) that range in size from 10 to 1000 nm with a substantial surface-to-volume ratio and significant functional surface have provided alternative innovative solutions for a variety of biomedical applications. Metal NPs, such as gold nanoparticles (AuNPs), iron oxide (FeO), fullerenes, carbon nanotubes (CNTs), titanium oxide nanoparticles (TiO_2_), zinc oxide nanoparticles, and silica nanoparticles have been used as therapeutic agents, or as carriers for drug molecules and diagnostic agents [12,13]. Silver nanoparticles (AgNPs) are among the most utilized metal NPs both in consumer products and in biomedical applications. AgNPs combine several advantages as size-dependent optical and physicochemical properties, a straightforward method of synthesis, a simple way to change their morphology and high surface area/volume ratio. AgNPs can be produced through different synthetic strategies, including chemical, thermal, irradiation, and green methods [14]. Nowadays, AgNPs are found in food containers, textiles, paints, and functionalized nanoparticles. In addition, they are widely used in medical practice in dental application and in wound healing dressings [15]. It is estimated that over 320 tons of AgNPs, with controlled size and geometry, are produced every year for biosensing, nanomedical imaging, and food applications [16,17]. Many studies have reported the antimicrobial activity of AgNPs, which had been attributed to the effect of released silver ions on bacterial cell walls, leading to changes in their membrane permeability. Further, intracellular interaction of silver ions can lead to the inactivation of sulphur and phosphorus-rich enzymes, affecting bacterial cell metabolism, reproduction, and viability [18,19]. While a large volume of literature exists on the antimicrobial effect of AgNPs on bacteria, a smaller number of studies are available regarding the effect of those metal NPs on human tissues, mostly related to their anticancer cytotoxic activity on various tumor cell lines [20]. Conflicting data exist from limited studies on the effect of AgNPs on the nervous system. For instance, Alon et al. [21] reported that AgNPs prompt the development of neural cells grown on an AgNPs-coated surface. However, other reports contradict this effect and suggest a toxic effect on human glioblastoma T98G as a model of glial cells in the central nervous system [22]. Furthermore, there is lack of uniformity regarding the dose and the characteristics of AgNPs reported in those conflicting studies. AgNPs can possibly be beneficial in wound dressings not only due to their antimicrobial activity, but also due to their anti-nociceptive action. In this study, we have investigated the anti-nociceptive of AgNPs. AgNPs were synthesized and characterized for their concentration and size. The anti-nociceptive effect of the prepared nanoparticles was measured using white albino rats injected with various doses of AgNPs and challenged using a hot-plate test and paw withdrawal latency (PWL). The chronic constriction injury (CCI) model was utilized to evaluate the pedal withdrawal reflex and tail withdrawal reflex, while an electrophysiological study was conducted utilizing colon longitudinal muscle strips.

## 2. Results

### 2.1. AgNPs Preparation and Characterization

**Figure 1:**
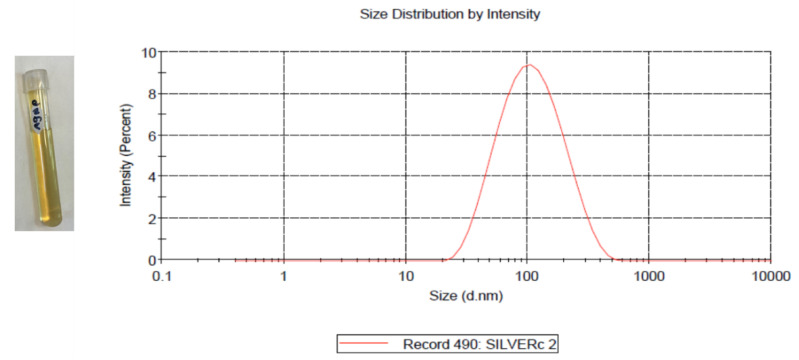
Prepared AgNPs (**left**); the average size AgNPs was 80 nm with a single peak at 123.5 nm, as measured by DLS (**right**).

AgNPs preparation followed the chemical reduction method using a AgNO_3_ aqueous solution as a seed, followed by utilizing tri-sodium citrate (Na_3_C_6_H_5_O_7_) as a reducing and capping agent. The reaction was performed at a high temperature to enhance the uniformity of the resulting AgNPs size. AgNPs showed maximum absorption at 431 nm. The resulting NPs had a concentration of 16 μg/mL and show an average size of 80 nm, as measured by dynamic light scattering (DLS) as shown in Figure 1.

### 2.2. Hot Plate Test

**Figure 2:**
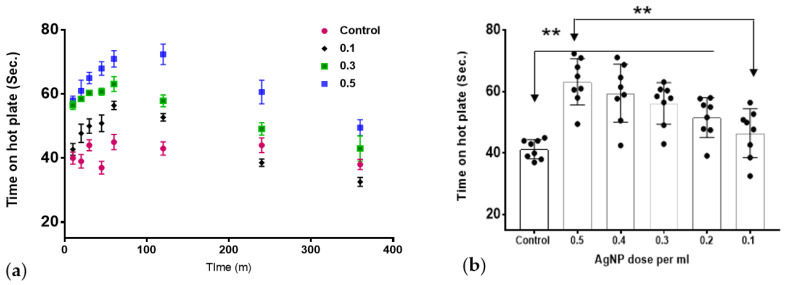
The effect of AgNPs on thermal withdrawal latency. (**a**) The temporal response over the study duration. (**b**) The overall thermal withdrawal latency across all time points. AgNPs showed significant increase in thermal withdrawal latency in treated groups (0.2–0.5 mL AgNPs) that showed a significant difference compared to the control group; the difference between the lowest dose and highest dose was also statistically significant (** *p* < 0.01) ”*n*” = 8.

The test was performed to detect the difference in response to heat between the AgNPs-treated rats and control rats. As shown in Figure 2a, the animals showed a higher tolerance to heat when treated with AgNPs compared to the control group. The effect was dose-dependent and peaks at around one hour after injection. The effect of the AgNPs was reversed and the animals showed a downward slope towards normal heat sensitivity at 4–6 h. Figure 2b shows the overall average difference for all the tested time points. 

### 2.3. Von Frey Hair Test

**Figure 3:**
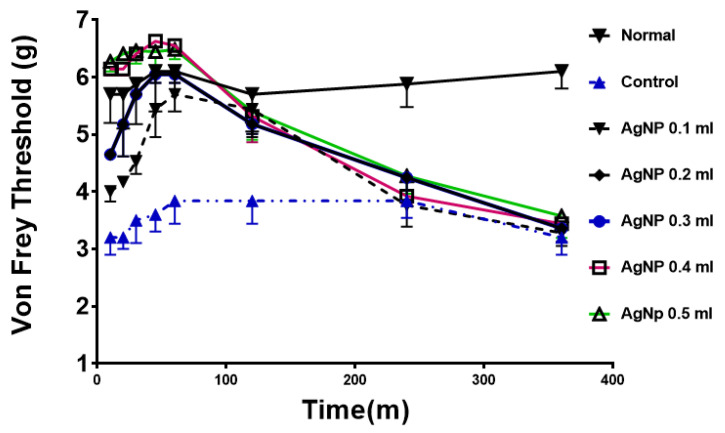
Anti-hyperalgesic effects of AgNPs treatment in rats. Normal; animals before constrictive surgery, control in blue; animals after surgery and before AgNPs treatment. All treated animals showed a significant difference compared to control group up to 2 h time point. (*p* < 0.05) “*n*” = 8.

The test was performed to test the effect of AgNPs on a model of chronic pain (chronic constriction injury model). Normal animals (prior to sciatic nerve ligation) show a high and consistent threshold to von Frey filaments. The control group (after surgery) shows a much lower threshold, consistent with hyperalgesia as a result of mechanical allodynia. Treatment with various doses of AgNPs (0.1 mL to 0.5 mL) showed a similar dose response towards normalizing the von Frey threshold. The effect is both time- and dose-dependent, as shown in Figure 3.

### 2.4. The Effect of AgNPs on Isolated Colon Muscle Strips

**Figure 4:**
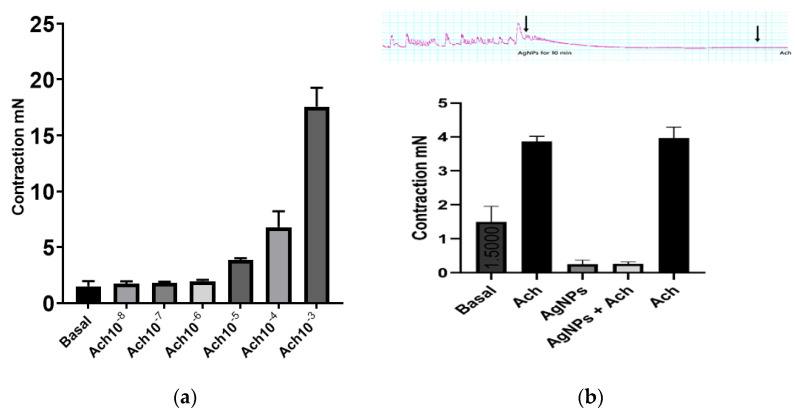
AgNPs abolished colonic migrating motor complexes (MMCs) and acetylcholine (Ach)-induced contractions. (**a**) Ach produced concentration-dependent contraction in the range of concentration used (10^−8^ M to 10^−3^ M). (**b**) Upper panel, AgNPs (8 µg/mL) abolished MMCs colonic activity in a time-dependent manner and prevented Ach-induced contraction; B lower panel, summary of data of the effect of AgNPs on MMCs and Ach-induced at Ach (10^−5^ M) contraction.

In mice colon longitudinal muscle strips, acetylcholine (Ach) caused a concentration-dependent contraction in the range of concentration used (10^−8^ M to 10^−3^ M), similar to the well-known effect of cholinergic agonists on GI smooth muscle (Figure 4a). AgNPs significantly abolished colonic smooth muscle basal activity (colonic migrating motor complexes (MMCs)) and the inhibitory effect was time-dependent, such that it reached complete inhibition 3 min after introducing AgNPs to the bath (Figure 4b upper panel). 

Ach at 10^−5^ M was unable to induce any change in muscle strip tone after 10 min incubation with AgNPs (Figure 4b). Interestingly, a test dose of Ach produced normal contraction after washing the organ bath three times, indicating that the muscle strips were viable and that reversible effect was due to the incubation with AgNPs.

## 3. Discussion

AgNPs are some of the most abundant nanoparticles that found their use in everyday products. It was estimated that >1620 products are identified as AgNPs-containing materials [23]. Different physical and chemical properties render appealing the use of AgNPs in comparison to their bulk analog. One of the most representative advantages is the small size and, consequently, the vast surface area of these nanomaterials. In addition, the ease of synthesis and production together with their high stability afforded a new generation of commercial products that, in turn, intensified scientific research in the nanotechnology area. AgNPs are of specific importance in many fronts due to their widespread usage. In medicine, AgNPs have been utilized in dental applications as well as in wound dressings due to their antibacterial activity [15]. In cancer research, AgNPs had been studied either as plain particles or as functionalized nanoparticles to target tumor cells [20]. Nonetheless, the systemic application of AgNPs is not currently approved since they are non-biodegradable and their long-term toxicity on different body systems is not well established. While the predominant characteristic of AgNPs is their antimicrobial actions, different studies emerged suggesting an effect on neuronal cells. These reports remain conflicting in their findings and utilized different dose ranges, sizes, and synthetic pathways. Liu et al. reported a dose-dependent effect of AgNPs on the level of LDH in embryonic neural stem cells, dose range (1–20 µg/mL); with a minimal effect at 1 µg/mL and the largest effect at 20 µg/mL [24]. In contrast, Gonzalez et al. reported a different and beneficial effect of AgNPs on N9 microglial cells. In their study, they indicated that AgNPs have a protective effect against neural damage at the dose of 50 μg/mL, through upregulating the cystathionine-γ-lyase enzyme responsible for producing hydrogen sulphide (H_2_S). H_2_S, in turn, results in the sequestration of the particles forming silver sulphide. Furthermore, the nanoparticles were protective against lipopolysaccharide (LPS)-stimulated ROS, nitric oxide, and TNFα production [25]. Furthermore, Ding et al. utilized the AgNPs scaffold at 2 mg/mL to promote nerve regeneration and its functional recovery in the animal model [26]. Relative to the current report, Liu and colleagues reported an inhibitory effect of AgNPs on voltage gated sodium channels in hippocampal CA1 neurons at a concentration of 10 µg/mL [27]. A similar finding was also reported by Strickland et al., who showed that AgNPs had an inhibitory effect of on pharmacologically induced neural network function in rat primary cortical cells [28]. In our current work, we tested the hypothesis that AgNPs could produce an anti-nociceptive effect in animal models of acute pain (hot-plate test) and in chronic pain (constrictive pain model). As shown in Figure 2, in the hot-plate test, the AgNPs showed a significant increase in the response time, which was a dose-dependent effect from 0.1 mL to 0.5 mL administration. The effect was time-dependent with a reversible nature that peaked at 60 m and back to base line at 4–6 h. The same pattern was observed in the von Frey test in a chronic pain model. Further, in an isolated colon muscle strip, a similar pattern arises with the inhibition of muscle tone either alone or in the presence of Ach. However, after washing the preparation to clear up AgNPs, the muscle strip was responding normally to Ach stimulation (Figure 4). This reproducible pattern further indicates the functional preservation of the muscle strip after exposure to AgNPs. Colonic migrating motor complexes (MMCs) are spontaneous cyclic contractions of neurogenic origin that are commonly seen in the isolated mouse colon muscle strip preparation. Application of a sodium channel blocker such as tetrodotoxin completely inhibited these cyclic contractions, supporting the neuronal origin of MMCs [29]. Interestingly, AgNPs have been shown to inhibit voltage-gated sodium channels in rat neuronal cells [27]. Thus, it is plausible to speculate that AgNPs in this study abolished MMCs by blocking sodium currents in neuronal cells that mediate these cyclic contractions. Taking all the results together, AgNPs could have a temporal inhibitory effect on nerve conduction. The potential use of AgNPs for pain relief should be guided by the in vivo safety of AgNPs. While the current study utilized a dose range of 1.6–8 micrograms of AgNPs on rats, several studies in animals had demonstrated that the toxic dose would be above 10 mg/Kg. The toxicity as well would depend on the route of administration, as AgNPs bioavailability varies based on the route of exposure. A comprehensive review of AgNPs by the European Commission Scientific Committee on Emerging and Newly Identified Health Risks (SCENIHR) reported the no observable adverse effect level (NOAEL) for AgNPs to be 30 mg/kg for a 90-day successive oral administration based on liver toxicity [30]. A recent study by Shehata et al. showed that a dose of 50 mg/kg daily oral administration of AgNPs for 90 days was needed to produce oxidative damage in renal and hepatic tissues [31]. While this report did not examine the mechanistic details of this effect, our results provide the first report on the possible use of AgNPs for managing local pain. Further work will be needed to elucidate the mechanism of the noted effect and its possible application, as well as an appropriate route of administration.

## 4. Materials and Methods

### 4.1. Synthesis and Characterization of AgNPs

Silver nanoparticles were prepared through a chemical reduction method, as described earlier [32]. In brief, 1 mM of silver nitrate solution was prepared in DW and heated to 95 °C. Then, 1% tri-sodium citrate was added and the solution was kept under vigorous stirring for 7 min. The solution was then cooled in ice. The resulting NPs were collected by ultracentrifugation at 27,000× *g* for 15 min using (Beckman J2-MC/JA-20). The supernatant was decanted and replaced with DW and then, centrifuged three times at 31,000× *g* for 20 min (Beckman L-80/NVT-65). The size and concentration were measured using a Malvern ZEN3600 Zetasizer Nano series (Malvern Instruments Inc., Westborough, MA, USA). 

### 4.2. Animals

A total of twenty-four male white albino rats with an average weight of (200 ± 50 gm) were included in this study. The animals were acquired from the National Research Center, Cairo. The animals were housed at a controlled temperature and were kept with free access to a standard rat chow diet and tap water. They were left for acclimatization for one week before the start of the study. The care of the animals before and during the experimental procedures was conducted in accordance with the guidelines of the Animal Ethical Committee, Faculty of Medicine, Suez Canal University. The study was approved by the Animal Research Ethics Committee of the college.

### 4.3. Hot Plate Test

The rats were placed on the hotplate at 50 ± 1 °C one at a time. The latency period for response (e.g., shaking, licking of the hind paw, or jumping off the plate to avoid the thermal stimulus) was recorded as response time. Rats were removed from the hotplate immediately after a response was observed. Thermal withdrawal latency was measured at multiple time points (10 m, 20 m, 30 m, 45 m, 1 h, 2 h, 4 h, and 6 h) after AgNPs administration. Treatment of the same group of AgNPs, Sc injection bilaterally on the dorsum of both paws at a Sc injection of 0.1 mL to 0.5 mL (e.g., 1.6 µg to 8 µg AgNPs), was conducted in consecutive days.

### 4.4. Chronic Constriction Injury Model

Animals were induced under ketamine anesthesia. The operation commenced after the loss of the pedal withdrawal reflex and tail withdrawal reflex. The surgical site on the left hind limb of the animals was shaved, sterilized, and marked. Incisions were made using a scalpel through the superficial fascia, exposing the biceps femoris. The pars cranialis and pars caudalis of the biceps femoris were separated to expose the underlying sciatic nerve. Once exposed, sciatic nerves were loosely tied 2 mm apart around the exposed nerve, with no occlusion of the nerve process. The nerves were gentled back into position under the biceps femoris. The wounds were closed and the skin was stapled. The animals were then allowed to recover and treated for post-operative pain. Treatment of the same group of AgNPs on the ipsilateral paw at 0.1 to 0.5 mL (e.g., 1.6 µg to 8 µg) was conducted in consecutive days. 

### 4.5. Von Frey Hair Test

The test was performed for testing mechanical allodynia; the test was undertaken 1 day prior to surgery (normal) and at 14 days post-surgery. The paw withdrawal threshold of the ipsilateral hind paw was measured immediately prior to AgNPs administration (control) and at multiple time points (10 m, 20 m, 30 m, 45 m, 1 h, 2 h, 4 h, and 6 h) after drug administration. Prior to testing, the animals were placed on an elevated wire mesh flooring (1.5 mm bars, 14 mm spacing) and contained with a mesh wire cylinder. The animals were habituated for at least 15 min in a darkened room. To calculate paw withdrawal thresholds, a logarithmically graded series of von Frey hair monofilaments (North Coast Medical Inc., Morgan Hill, CA, USA) were presented to the left hind paw. The hairs presented had the following force intensities: 3.2, 3.84, 4.17, 4.56, 5.07, 5.88, 6.1, 6.45, and 6.65 gm/mm. Monofilaments were applied to the left ventral plantar aspects perpendicular to the hind paw for 3 s. A withdrawal was considered as a paw flinch upon presentation of a given monofilament.

### 4.6. Electrophysiological Study

Muscle strips were prepared from the colon of Balb/C mice after CO_2_ euthanasia. The colon was removed, flushed with Krebs buffer, and placed in warmed (37 °C) Krebs buffer of the following composition (in mM): 118 NaCl, 4.75 KCl, 1.19 KH_2_PO_4_, 1.2 MgSO_4_, 2.54 CaCl_2_, 25 NaHCO_3_, 11 mM glucose (pH 7.4), and bubbled with 95% O_2_/5% CO_2_. Muscle strips were tied in the orientation of the longitudinal muscle layer at both ends with surgical silk. One end was tied to the force transduces of a DMT 720MO organ bath system. The other end of the muscle strip was tied to a hook and placed in the organ bath horizontally. The bath was filled with 5 mL of warmed Krebs buffer, and changed at 15 min intervals during equilibration and after each test drug. The force recordings were amplified using a DMT amplifier and the data acquisition was by an AD instrument lab chart 8 software. The strips were allowed to equilibrate at 1 g tension for at least 1 h before the experiment. Exposure to AgNPs and Ach was performed while the strips were suspended in the organ bath. Control responses to Ach were first established before the subsequent addition of test agent. Each muscle strip was challenged with a defined concentration of Ach for 3 m and then washed for a total each separated by 15 min. Then, a dose response curve for Ach was generated using Ach from 10^−8^ M to 10^−3^ M; a second cycle of three times wash was then applied and the strips were tested with AgNPs for 10 min; then, Ach 10^−5^ M was introduced to the bath for 3 min. Finally, after a third cycle of three times wash, the strips were tested with a defined dose of Ach for 3 min.

### 4.7. Statistical Analysis

All the data are presented as mean ± SD unless mentioned otherwise. The significance of difference between the groups was conducted using an analysis of variance (ANOVA test) by comparing the response in the control group vs. the AgNPs-treated groups.

## Data Availability

The data presented in this study are available on request from the corresponding author.

## Sample Availability

Samples of the compounds are available from the authors.
